# Enhancing Intraoral Scanning Accuracy: From the Influencing Factors to a Procedural Guideline

**DOI:** 10.3390/jcm14103562

**Published:** 2025-05-20

**Authors:** Anca Maria Fratila, Adriana Saceleanu, Vasile Calin Arcas, Nicu Fratila, Kamel Earar

**Affiliations:** 1 Faculty of Medicine, Lucian Blaga University of Sibiu, 550169 Sibiu, Romania; anca.fratila@ulbsibiu.ro; 2 Military Clinical Emergency Hospital of Sibiu, 550024 Sibiu, Romania; 3 Doctoral School, Lucian Blaga University of Sibiu, 550169 Sibiu, Romania; 4 Waterpoint Research, 550075 Sibiu, Romania; nicu.fratila@waterpoint.ro; 5 Faculty of Dental Medicine, “Dunarea de Jos” University, 800216 Galati, Romania; erar_dr.kamel@yahoo.com

**Keywords:** intraoral scanning accuracy, trueness and precision of intraoral scanning, influencing factors, scanning strategy, scanning procedural guideline, scanning protocol

## Abstract

**Background/Objectives**: Intraoral scanning, a fast-evolving technology, is increasingly integrated into actual dental workflows due to its numerous advantages. Despite its growing adoption, challenges related to the accuracy of digital impressions remain. The existing literature identifies most of the factors influencing intraoral scanning accuracy (defined by precision and trueness), but it is fragmented and lacks a unified synthesis. In response to this gap, the present study aims to consolidate and structure the current evidence on the determinant factors and, based on these findings, to develop a clinically applicable procedural guideline for dental practitioners. **Methods**: A comprehensive literature review identified 43 distinct factors influencing intraoral scanning. **Results**: These factors encompass variables such as software versions and updates, implant characteristics (e.g., position, angulation, scan body design), materials, environmental conditions (e.g., lighting), and procedural elements including scanning strategy, pattern, aids, and operator experience. Subsequently, these identified factors were systematically classified into five distinct groups based on inherent similarities and relevance within the scanning workflow: IOS—characteristics and maintenance, intraoral morphology, materials, ambient conditions, and scanning strategy. To translate these findings into a practical framework, a four-step protocol was developed, designed for straightforward application by researchers and clinicians. **Conclusions**: This protocol—comprising: (1) Maintenance, (2) Evaluation, (3) Establishment and Execution of Scanning Strategy, and (4) Verification—aims to guide users effectively through the intraoral scanning process, mitigate common clinical challenges, and ensure broad applicability across diverse scanner systems, irrespective of the manufacturer or model.

## 1. Introduction

The intraoral scanner (IOS), one of the latest innovations in digital dentistry [1], is widely adopted by clinicians [2] and proven to be a reliable alternative to conventional dental impressions [3]. Computer-aided design and computer-aided manufacturing technologies are enhanced due to the impression procedures [4] realized with IOSs [3]. IOS is widely applicable in prosthodontics, implantology, and orthodontics, while effective for single-unit and short-span restorations, accuracy may be compromised in extensive restorations [5].

The digital impression acquired with an IOS has multiple advantages such as: patient comfort [5,6,7], streamlined workflow [8,9], the ability to repeat scans [10] or partially rescan and overwrite specific areas [6,7,11], treatment planning with direct on-screen simulation [5,10], reduced transportation time and costs [6,12,13], minimized material waste [7,14], seamless data exchange with dental laboratories [15], enhanced infection control safety [15], improved airway safety during impression-taking [16], high accuracy [6,17], and integration with maxillofacial data [14]. At the same time, IOS also serves as a valuable marketing tool for dental practices [11].

Beside the advantages, it also confronts with challenges: high costs of hardware [5,18,19], lack of training offer [18], lack of know-how in opportunity of different scanning strategies [18], limited information offered by the manufacturers [18], lack of guidance in choosing suitable IOS [19], the necessity of upgrade the old workflow and procedures [16,20], the necessity of large data storages [16], decreased accuracy for complete-arch digital scans [5,19,21], difficulty in recording deep marginal lines of prepared teeth [5,22] and areas with high tissue mobility [23].

Currently, various technologies are available in iOS. Some of the technologies used are three-dimensional (3D) reconstruction, confocal imaging [24,25], active triangulation, optical coherence tomography, and active wavefront sampling [25,26,27,28], and some IOS uses more than one technology [29]. Every technology possesses unique strengths, and its accuracy is variably influenced by an array of factors [29,30,31,32,33]. This variability underscores our intention to establish a universally applicable set of best practices, irrespective of the type of IOS system in use.

IOS is an evolving device [11,34], the rapid and ongoing advancement of hardware and software of the IOS is notable [16,31]: haptic feedback for guided scanning was added, integrated heaters to mitigate condensation [16], fluorescent light that reveals oral bacteria on the tooth surfaces [24], detecting dental wear [35], faster scanning, autocalibration, powder-free scanning process, and color image acquisition [16] and overall the fact that their accuracy continues to improve over time [16,31].

The IOS market is dynamic due to the many manufacturers in the field, with continuous hardware and software updates, and the rapid pace of development that can improve performance [36,37,38]. It is not possible to give a precise number of available IOS models. We can estimate that more than twenty IOS models are commercially available in the world [14], or even more than 35 different systems [39]. Given the extensive array of commercially available IOS, this review will abstain from referencing specific brands or models. Instead, the analysis will concentrate solely on factors that have been proven to influence the accuracy of these systems.

Accuracy is defined by the standard ISO 5725-1:2023 using two terms at the same time: trueness and precision [40]. Trueness defines the ability of the IOS to reproduce a dental area as closely to its real form without errors [40,41]. Precision refers to the degree of reproducibility of the measurements, and in other words, how similar the digital impressions are if they are repeated more times [40].

The accuracy of the intraoral digital impression is critical [33] and determines not only the overall fit and quality of the restoration but also the lifespan of the prosthetic [33,42]. An inaccurate scanning process can lead to restorations that are not properly marginal and internally adapted [5,29], compromising periodontal health, functional efficiency, and the durability of the restoration, necessitating further clinical interventions [32].

We acknowledge a number of reviews examining the accuracy of intraoral scanning [5,6,11,23,25,29,32,33,34,35,36,43,44,45,46,47]. The literature that tries to consolidate most of the influencing factors is scarce and sometimes contradictory [48], however, there are some studies that have managed to compile in an extensive manner the numerous factors influencing IOS accuracy [32,33]. Nevertheless, as underscored in this study, our objective is not solely to reproduce prior studies but to furnish an integration of existing knowledge into a comprehensive framework, a practical guideline, for a standardized approach that leads to successful intraoral scanning.

The motivation behind this study stems from the growing adoption of IOSs in digital dentistry and the need for a comprehensive, standardized approach to optimizing their accuracy. Given the rapid advancements in IOS hardware and software, along with the variability in scanning outcomes due to environmental, technical, and operator-related factors, there remains a critical gap in consolidated, evidence-based guidelines. This study aims to bridge that gap by integrating existing research into a structured framework, providing clinicians with practical insights to enhance the accuracy, reliability, and efficiency of intraoral scanning procedures.

## 2. Materials and Methods

### 2.1. Literature Search Strategy and Selection Criteria

The search was conducted between January 2019 and December 2024, focusing on peer-reviewed publications related to the accuracy of IOS. To enhance the reliability of the selection process, three independent researchers (AMF, VCA, NF) were assigned specific tasks: one researcher formulated the search queries and applied inclusion/exclusion criteria, another reviewed and screened the retrieved articles for relevance, and the third cross-verified the selected studies and extracted key data. This multi-step approach ensured an unbiased synthesis of the existing literature, leading to the identification of studies that provide valuable insights into the factors influencing IOS accuracy.

The literature search was conducted following PRISMA 2020 guidelines, ensuring a systematic and transparent methodology [49]. The search was focused on the accuracy, precision, fidelity, and trueness of IOS. The Scopus, PubMed, and Web of Science (WOS) were explored. The Scopus database was queried using the following search: “TITLE (intraoral AND scanning AND (precision OR accuracy OR fidelity OR trueness)) AND NOT TITLE (cbct) AND PUBYEAR > 2018 AND PUBYEAR < 2025 AND (LIMIT-TO (LANGUAGE, “English”)) AND (LIMIT-TO (OA, “all”))” [50]. The PubMed database was queried using: “(((intraoral [Title] AND scanning [Title] AND (precision [Title] OR accuracy [Title] OR fidelity [Title] OR trueness [Title]) NOT (cbct [Title])) AND ((“2019” [Date-Publication]: “2024” [Date-Publication]))) AND (English [Language])” and additionally the “Free full text” was selected box. The WOS database was queried using the following: “intraoral AND scanning AND (precision OR accuracy OR fidelity OR trueness) (Title) not cbct (Title)” and additionally the English language was selected, the period, and “All Open Access”. This strategy aimed to exclude studies unrelated to intraoral scanning, particularly those focusing on CBCT technology, and to prioritize research published within the last six years. The inclusion criteria focused on open-access, English-language articles published between 2019 and 2024, specifically addressing factors influencing IOS accuracy. Studies that lacked empirical data or did not provide quantitative assessments of IOS accuracy were also removed. Non-English publications, conference abstracts, case reports, editorial opinions, and letters to the editor were not considered. Additionally, articles with small sample sizes, or with research processes incomplete or insufficiently described to assess replicability, or lacking statistical validation, were excluded to maintain the study’s scientific rigor. By applying these criteria, only the most relevant and high-quality research was included in the final review.

**Figure 1:**
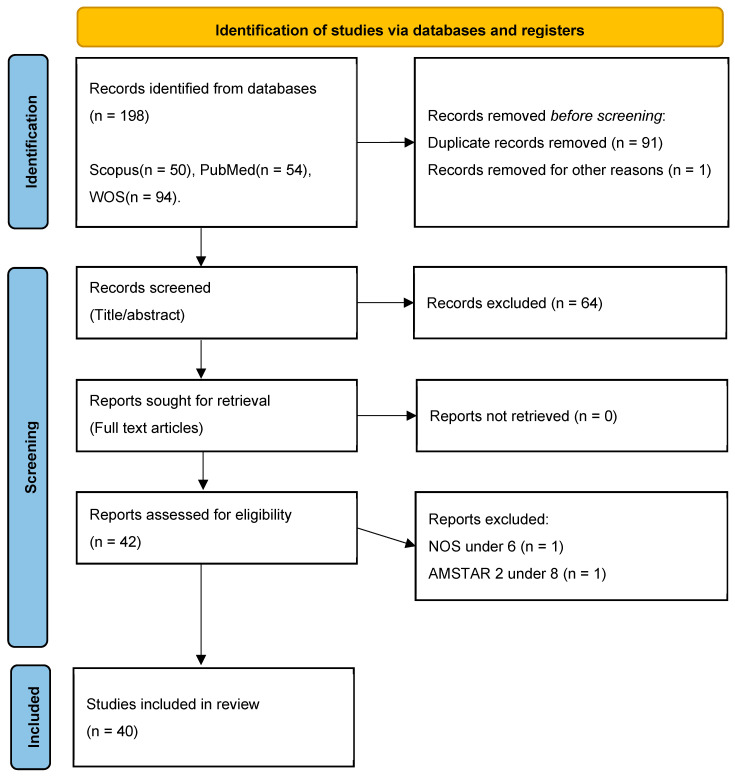
PRISMA flow chart, illustrating the process of choosing articles to be included in this study, based on the standards of Kahale et al. [49].

Initially, 106 articles were retrieved, and after screening for relevance, 42 studies were selected for final analysis as seen in Figure 1. This rigorous keyword-based approach ensured the inclusion of high-quality evidence to support the study’s objectives.

The selection of relevant articles followed the PRISMA protocol, ensuring a systematic and transparent review process. Initially, articles were retrieved based on their titles, and any duplicate entries were removed to avoid redundancy. Next, the remaining studies were screened by abstract, and those that were not conclusive or did not directly address the accuracy of IOSs were excluded. Following this, the full-text articles of the remaining studies were sought for retrieval. To ensure the inclusion of high-quality and methodologically sound research, the studies were then assessed for eligibility using the Newcastle–Ottawa Scale (NOS) for observational studies and AMSTAR 2 tool for reviews and meta-analyses [51,52]. Only those articles that met the predefined quality criteria were included in the final synthesis, ensuring that the study was built on robust and credible scientific evidence, therefore, only studies with a Newcastle–Ottawa Scale (NOS) score of 6 or higher, or an AMSTAR 2 rating greater than 8, were included. A detailed overview of this process is presented in the PRISMA flowchart above, visually illustrating each phase of article selection, from identification to final inclusion.

### 2.2. Registration

This study has been registered on the Open Science Framework (OSF) to ensure transparency, reproducibility, and accessibility of the research process. The registration includes the full study protocol, search strategy, inclusion/exclusion criteria, and extracted data, allowing other researchers to review and validate the methodology. By making this information publicly available, we aim to contribute to open science practices and support further advancements in intraoral scanning accuracy research. The complete protocol and extracted data can be accessed via the following link: https://osf.io/n8pwd.

### 2.3. Data Extraction and Quality Assessment

The data extraction strategy was designed to ensure consistency, accuracy, and completeness in gathering relevant information from the selected studies. A standardized data extraction form was developed, which included key variables such as study title, authors, year of publication, study design (in vitro or in vivo), influencing factors, and key findings. Each selected article was independently reviewed by two researchers (AMF, VCA), who extracted the relevant data according to predefined criteria. To minimize bias and ensure data integrity, the extracted information was cross-checked among the researchers. Any disagreements among the reviewers were resolved through discussion; if consensus could not be reached, an impartial third-party reviewer (NF) was consulted to make the final decision. This approach aligns with best practices in systematic reviews, where involving an additional reviewer helps to minimize bias and enhance the accuracy of data extraction.

The quality assessment of the selected studies was conducted using the Newcastle–Ottawa Scale (NOS) for observational studies [51]. The NOS scale was used for observational studies, scoring them based on selection criteria, comparability, and outcome assessment to determine their reliability. Each study was independently assessed by the three researchers.

The NOS was employed to systematically assess the quality of observational studies included in this review, ensuring methodological rigor and reliability. This tool evaluates studies based on three primary domains: selection of study participants, comparability of study groups, and outcome assessment. The selection domain examines the adequacy of case definition, representativeness of the sample, and ascertainment of exposure. The comparability domain assesses whether the study controls for key confounding factors, ensuring that differences in outcomes are due to the variables being studied rather than external influences. Lastly, the outcome domain evaluates the methods used to assess results, the length of follow-up, and whether follow-up was sufficient to ensure meaningful conclusions.

Each study was scored on a nine-point scale, with a higher score indicating superior methodological quality. To maintain the validity and credibility of the review, only studies that achieved a score of six or higher were included. Studies with scores below this threshold were excluded to prevent the incorporation of research with potential biases, inadequate control of confounding variables, or insufficient outcome assessment.

In this study, the methodological quality of included systematic reviews was assessed using the AMSTAR 2 [52] (A Measurement Tool to Assess Systematic Reviews 2) checklist. AMSTAR 2 is a comprehensive and validated critical appraisal tool designed to evaluate the reliability and methodological rigor of systematic reviews that include both randomized and non-randomized studies of healthcare interventions. The instrument comprises 16 items, of which seven are considered critical domains, including protocol registration, adequacy of the literature search, justification for excluding studies, risk of bias assessment, appropriateness of meta-analytical methods, consideration of risk of bias when interpreting results, and assessment of publication bias.

Based on the evaluation, reviews were rated as high, moderate, low, or critically low quality. For the purposes of this analysis, only systematic reviews that achieved a score greater than 8 were considered to be of sufficient methodological quality and were thus included in the final synthesis. This threshold was chosen to ensure a high standard of evidence reliability and to minimize the risk of bias in the conclusions drawn from the included literature.

This structured evaluation process ensured that only methodologically sound, high-quality systematic reviews and meta-analyses were included in the final synthesis, strengthening the reliability and validity of the study’s conclusions.

### 2.4. Data Synthesis and Analysis

The data synthesis process involved systematically organizing and analyzing the extracted data to identify trends, patterns, and key findings related to the factors influencing the accuracy of IOS. The synthesis was conducted in a narrative format for qualitative data, summarizing the various influencing factors. Studies were grouped based on methodological design (in vitro vs. in vivo) to facilitate a structured comparison. To ensure consistency, standardized effect measures such as mean differences, standard deviations, and confidence intervals were extracted where applicable. The findings were then synthesized to establish a comprehensive framework of best practices for improving IOS accuracy.

## 3. Results

### 3.1. Databases Research Results and Validity of Data Extraction Assessment

The database research process yielded a total of 198 records identified across the following databases: Scopus (50), PubMed (54), and WOS (94). Before screening, 91 duplicate records were removed, along with 1 additional record excluded for other reasons. This resulted in 106 records that proceeded to the title and abstract screening phase. At this stage, 64 records were excluded due to a lack of relevance or failure to meet the predefined inclusion criteria. The remaining 42 articles were then sought for full-text retrieval.

The methodological quality of the studies included in this investigation on enhancing intraoral scanning accuracy was rigorously assessed using two standardized appraisal tools: the Newcastle–Ottawa Scale (NOS) for observational studies and the AMSTAR 2 checklist for systematic reviews. These tools enabled a structured evaluation of study design, methodological rigor, and overall risk of bias. To ensure that only high-quality evidence informed the synthesis and conclusions, predefined thresholds were set: a minimum NOS score of 6 and an AMSTAR 2 total score of 8.

Out of the 38 observational studies evaluated using the NOS, 37 met or exceeded the minimum quality threshold. This indicates that nearly all studies demonstrated adequate methodological soundness in terms of cohort selection, comparability, and outcome assessment. Only one study was excluded for failing to meet the minimum score, reinforcing the general reliability of the observational evidence base supporting this review.

The methodological appraisal using the Newcastle–Ottawa Scale (NOS) revealed that among the 38 observational studies assessed, the majority achieved high-quality scores. Specifically, 16 studies obtained a score of 9, indicating excellent methodological rigor. Fifteen studies scored 8, and six scored 7, all surpassing the threshold of 6. Only one study received a score of 5 and was excluded from further analysis due to insufficient methodological quality.

In contrast, the quality of systematic reviews assessed via the AMSTAR 2 tool showed greater variability. Of the four reviews analyzed, three achieved a score of 8 or higher and were included in the final analysis. The other one was excluded due to lower scores, often stemming from deficiencies in protocol registration, inadequate risk of bias assessment, or limited methodological transparency in meta-analytical techniques. One review received a score of 7, falling just below the inclusion threshold. In contrast, two reviews scored 13, and one scored 14, demonstrating strong adherence to methodological standards. These three high-scoring reviews were deemed suitable for inclusion.

This selective inclusion process ensured that the evidence base used to develop procedural guidelines was robust and credible. By excluding lower-quality sources, the study mitigated potential bias and enhanced the validity of its findings and recommendations. This rigorous approach underscores the importance of critical appraisal in evidence-based practice, particularly in the evolving domain of digital dentistry and intraoral scanning technologies. This scoring distribution highlights a strong foundation of quality within the observational studies informing this research, contrasted with a more limited pool of reliable systematic reviews. The predominance of high-scoring observational studies strengthens the empirical basis of the findings, while the selective inclusion of only the most rigorous reviews further supports the credibility of the synthesized evidence.

**Figure 2:**
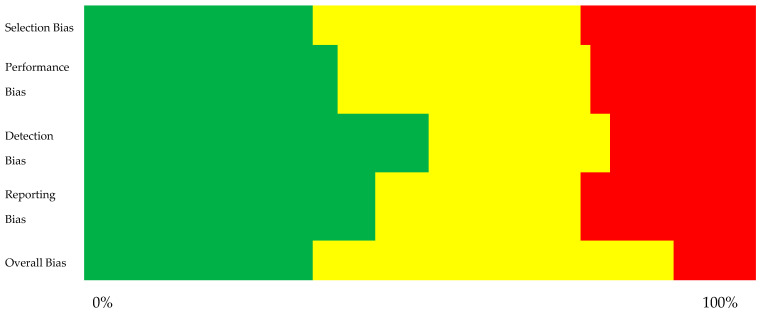
Summary plot illustrating the risk of bias across different aspects (green – low risk of bias, yellow – moderate risk of bias, red – high risk of bias).

The risk of bias assessment was a critical step in ensuring the reliability of the included studies. Studies with high risks of bias, such as inadequate participant selection, lack of control for confounding factors, or poor reporting transparency, were either excluded or flagged for cautious interpretation. The risk of bias assessment was conducted by categorizing each study into four key domains: selection bias, performance bias, detection bias, and reporting bias. Each domain was rated as having a low, moderate, or high risk based on the overall methodological quality indicated by the NOS for observational studies as seen in Table 1 and AMSTAR 2 for systematic reviews as seen in Table 2. The overall risk of bias was determined by the most frequently occurring level across these domains for each study, this can be visualized in Figure 2.

By implementing these assessments, this study mitigated potential biases, ensuring that the conclusions were based on high-quality, methodologically sound research with minimal risk of distortion. Overall, the dual-layered approach to risk of bias assessment ensured that the body of evidence underpinning the study’s conclusions was methodologically sound and credible.

### 3.2. Literature Findings

Table 3 provides an overview of the factors identified in the literature as influencing the accuracy of IOSs. Each factor is associated with the corresponding research reference and a specification of whether the study was conducted in vitro or in vivo. This synthesis enables a structured understanding of the various factors influencing IOS accuracy, as demonstrated by empirical research.

Further investigation beyond the initial set of articles revealed additional factors influencing IOS accuracy not initially identified (Table 4).

A detailed analysis was conducted on the identified factors in order to derive an intuitive framework that is both intuitive and easily understood and readily applicable. These factors have been systematically categorized into five distinct groups based on inherent similarities and relevance within the scanning workflow:

1. **IOS—characteristics and maintenance**—this includes the inherent features of the IOS device, its pre-scanning preparation, and ongoing maintenance protocols.
2. **Intraoral morphology**—this category includes the anatomical characteristics of the oral cavity and dentition.
3. **Intraoral materials**—this refers to the presence of various materials within the oral cavity, such as restorative or implant scan body materials.
4. **Ambient conditions**—this category encompasses the environmental conditions under which the scanning procedure is performed, including wetness, lighting, and temperature.
5. **Scanning strategy**—this refers to the specific techniques and approaches employed by the operator during the scanning process.

Through a detailed analysis of each factor group mentioned previously, and by consolidating factors with different names but referring to the same fundamental aspect, the following table (Table 5) has been constructed. This table provides a clear and concise overview of the main factors influencing the accuracy of IOSs.

## 4. Discussion

### 4.1. IOS—Characteristics and Maintenance

#### 4.1.1. Software Versions and Updates

While the impact of hardware on IOS performance is well-established, the influence of software remains relatively under-investigated [79]. Recent studies indicate that software updates can significantly improve the accuracy of digital impressions [79,80]. Schmalzl et al. (2023) observed an association between recent software versions and improved accuracy, but emphasize that the relationship is not linear and the impact of updates varies [68,79]. Vág et al. (2021) confirm these variable effects; some scanners show significant improvement, while others experience reduced accuracy [80]. Zarauz et al. (2023) found that newer software versions for the tested IOSs improved the trueness of scans performed by inexperienced operators, but did not significantly affect the accuracy of experienced operators [68]. These findings highlight the importance of considering software versions as a critical factor in evaluating IOS performance [68,80,81]. For practitioners, it is recommended to evaluate the necessity of updates and to integrate the update procedure into their workflow [80]. The practitioner must also take into account the version of the CAD/CAM software utilized. It has been validated that more recent software can enhance the precision of restorations [82].

#### 4.1.2. Scan Resolution

While resolution is considered a desirable feature of IOS [5], and is less frequently studied [83], contrary to expectations, several studies have found no significant relationship between resolution and the accuracy of digital impressions. Chiu et al. (2020) observed no significant difference in accuracy between default and high-resolution scans [84]. Similarly, Medina-Sotomayor et al. (2018) reported no correlation between resolution and accuracy [85]. In a clinical context, the usage of the high-resolution IOS function implies an increased duration of scanning [84].

#### 4.1.3. Size of the Scanner Head

The size of the IOS head influences both accuracy and practicality. While smaller tips offer improved access in posterior regions or for patients with limited mouth opening [5,86], they can negatively impact trueness and precision [86]. This is attributed to the smaller field of view, necessitating more image superimpositions to create a complete 3D model [86]. As a counterpoint, larger scanning heads demonstrate superior trueness and precision due to their wider imaging range, which requires fewer scans and minimizes camera-shaking errors [32,87]. However, larger tips may present challenges in certain clinical situations. Ultimately, the choice of tip size should be guided by the specific clinical context and the required level of accuracy. While small tips may be suitable for applications where precision is less critical, such as diagnostic casts or orthodontic aligners [86], their use should be limited when high accuracy is paramount, especially for full-arch impressions [86].

#### 4.1.4. Custom Abutment Library Data

Accurate intraoral scanning for dental implants requires the management of the following challenges: an inaccurate scan may result because of a subgingival location or restricted accessibility [88], the soft tissue can collapse during connection of the scannable abutment [89], the intraoral scanning does not record the margin exactly due to the reflexive surface of the abutment, the scanning requires more gingival displacement [88], the risk of various side effects caused by using tissue displacement cords [90] or the space between the abutment and adjacent teeth is narrow [88]. To overcome these difficulties, a technique involving the superimposition of prescanned abutment data is proposed to improve the accuracy of digital scanning [88,90]. The digital library of custom abutments can be obtained by scanning in the lab [90] or from the CAD software [89]. This technique allows for the rapid fabrication of an accurate prosthesis [89,91].

#### 4.1.5. Calibration

Maintaining the IOS requires diligent calibration. Calibration of the IOS has received limited attention in the literature, and it is absent from the list of factors influencing the accuracy. Calibration is essential to ensure the reliability of IOS systems [36,92], with significant reductions in accuracy observed when calibration is neglected [36]. Following manufacturer recommendations for calibration is considered best practice and is essential for ensuring the accuracy and reliability of IOSs [38,62,72,92]. This includes performing calibration before each scan, particularly when ambient temperature fluctuates [72,92], as environmental changes can significantly impact scanner accuracy [92]. Calibration procedures may encompass both 3D and color calibration [72], and optimal parameters may vary for individual IOS systems [32]. Abduo (2018) reported a significant positive effect of manufacturer calibration on the trueness of certain IOS systems [36]. Ultimately, proper IOS calibration is critical for obtaining accurate digital models and ensuring the clinical success of restorations [62]. However, the lack of standardized calibration procedures and the variability in methodologies across studies highlight the need for universally accepted guidelines for IOS accuracy evaluation [36].

### 4.2. Intraoral Morphology

#### 4.2.1. Crowding and Inclination of Teeth

Martínez-Rodríguez et al. (2020) [1], evaluating the accuracy of scanning in the context of increasing amounts of crowding and with a significant molar inclination, have identified greater amounts of undetected volume and scanning failures. Based on these results, we can state that severe crowding and considerably angled molars near an edentulous gap show a statistically significant loss of accuracy [1].

#### 4.2.2. Edentulous Condition

The extent of edentulism impacts the accuracy. Studies by Lee et al. (2021) and Jin et al. (2021) have shown that as the number of missing teeth increases, the precision of IOS diminishes [9,58]. This is particularly evident in edentulous regions with more than two missing teeth, where the registration of occlusal relationships becomes notably inaccurate [58]. These findings underscore the importance of considering the span and location of the edentulous condition when utilizing IOS for digital dental impressions, especially in cases of extensive tooth loss [9,58].

#### 4.2.3. Palatal Morphologies

In the context of an edentulous maxillary, the buccopalatal morphology represents a factor in the accuracy of intraoral scans. The literature on buccopalatal morphology is limited, with most insights from Sorrentino et al. (2024) [69]. Optimal scan accuracy was observed in medium palatal depths, with no differences between flat and deep palates. Significant inaccuracies occurred at the base of the palatal vault and maxillary tuberosities in deeper palates. Despite these localized deviations, the overall mean trueness and precision remained clinically acceptable for removable denture fabrication. Palatal rugae showed no effect on scan accuracy across morphologies [69]. Ruggiero et al. (2024) [93] present findings that reinforce some of the previously mentioned observations. The highest scan accuracy was associated with medium palatal depth, and mean accuracy values for all palatal morphologies remained within clinically acceptable limits as reported in the literature. No consistent evidence was found that palatal rugae either enhanced or impaired scan accuracy across the morphologies evaluated. Nevertheless, in deeper palates, the presence of rugae contributed to improved scan precision. Furthermore, in flat palatal configurations, trueness was superior when rugae were present compared to when they were absent [93]. Arch width was shown to influence the precision of intraoral digital impressions in fully dentate cases, with decreased precision associated with increased arch width [94]. Zarone et al. (2020) [95] evaluated several scanning strategies, identifying the buccopalatal (BP) technique as the most accurate. This method involves initiating the scan at the crest of the edentulous ridge, beginning at the left maxillary tuberosity and progressing longitudinally along the ridge to the right tuberosity. The scan then continues along the buccal aspect, followed by the palatal vault. The palatal region is first captured using a counterclockwise motion along the vault, concluding with a longitudinal posteroanterior pass to close the midline gap of the palate [95].

#### 4.2.4. Inter-Tooth Distance

Spatial constraints between a prepared tooth and its adjacent structures have been shown to interfere with the intraoral scanning process, potentially limiting the capture of accurate surface data. However, evidence suggests that when the interproximal distance exceeds 1.5 mm, scanning accuracy is not adversely affected and may, in fact, improve [96]. Optimal accuracy has been reported when the spacing reaches or exceeds 2.0 mm, facilitating unobstructed scanner access and more reliable digital impressions [73]. For more than 3.5 mm distance, there was no significant improvement in accuracy reported [73]. Restricted interdental spacing can compromise intraoral scanning accuracy by limiting the accessibility of the scanner tip and constraining the range of scanning angles, thereby obstructing the precise digitization of surface morphology [48,97]. Such spatial limitations may also induce shadowing effects, leading to localized data loss within the scanned area [96]. In these instances, the scanner software may rely on interpolative reconstruction to compensate for missing data, a process that can introduce further inaccuracies into the digital impression [98]. Interdental spaces narrower than 0.5 mm present a considerable challenge for less experienced operators, often necessitating extended scanning durations and a higher number of photograms to achieve adequate data acquisition, in contrast to experienced clinicians who can perform the task more efficiently [99].

#### 4.2.5. Inter-Implant Distance

The inter-implant distance, defined as the distance between two adjacent implants, has been identified as a factor that may influence the accuracy of intraoral digital scans [48]. Although the current literature addressing this relationship remains limited [48], emerging evidence suggests that increased inter-implant spacing is associated with cumulative deviations in both linear and angular dimensions, likely due to errors accumulating during the digital image stitching process [97]. This phenomenon is further supported by findings indicating that greater Euclidean distances between implants negatively affect the trueness and precision of digital impressions [100]. Consequently, minimizing inter-implant distances may contribute to enhanced global linear accuracy by reducing distortion in IOS outputs [100,101]. The decline in trueness is frequently ascribed to the increased number of alignment steps required to generate a complete three-dimensional model, with the absence of stable reference landmarks between widely spaced implant scan bodies (ISBs) compromising the efficiency of the image stitching process [102]. Studies indicate that, although differences in accuracy metrics may reach statistical significance, they do not necessarily correspond to clinically relevant deviations that would adversely affect the fit or function of the definitive prosthesis [97,103].

#### 4.2.6. Implant Position

Gomez-Polo et al. (2022) [3] have conducted an investigation into the impact of implant positioning on the accuracy of the digital IOS scan. Their findings indicate that there is an influence of the implant position due to distortion during the stitching procedures, and additional studies are recommended to clearly elucidate this aspect [3].

#### 4.2.7. Implant Depth

Implant depth refers to the vertical positioning of a dental implant in relation to the adjacent mucosal tissues. Although research on this topic remains limited [48], several key findings have emerged: IOS generally demonstrate superior trueness and precision compared to conventional impression techniques for implant subgingival depths up to 6 mm [104] and the trueness and precision of scans are highest when the implant is positioned at the mucosal level, allowing full visibility of the ISB [105]. These parameters progressively decline with increasing subgingival placement. However, within the initial 3 mm of submergence, no statistically significant deterioration in scan accuracy has been observed [105].

#### 4.2.8. Implant Angulation

With regard to the angulation of implants, the findings are inconsistent; certain studies suggest that it does not exert an influence [48,106], while others assert that it does [44,48]. The article incorporated into our review concludes that the scanning accuracy decreases as the implant angulation increases, underscoring the necessity to attend to this particular factor [3].

#### 4.2.9. Implant Scan Body Fit

The accurate fit of ISBs is essential for the reliable transfer of implant position and angulation in digital workflows. Misfit may compromise prosthetic accuracy, particularly for angled implants, where rotational discrepancies can cause horizontal deviation [107]. The design of the implant–abutment index and manufacturing tolerances further influence the precision of the fit, underlining the need for improved precision by manufacturers [107]. Internal conical connections have been shown to result in greater axial discrepancies compared to external flat-to-flat interfaces, which offer more stable positioning [108,109]. Torque values also affect scanbody seating depth; higher torque in conical interfaces leads to deeper insertion and vertical shifts [108,109]. Variation in scanbody design—including length, diameter, and angle—exists even within components from the same manufacturer, impacting the transfer of implant position and contributing to misfit [110,111]. Repeated use and sterilization cycles can degrade the fit over time, reducing intraoral scanning accuracy [112]. Detachment and repositioning may cause further deviation, depending on the scanbody system [113,114]. Material choice also matters; metal scanbodies offer greater dimensional stability compared to PEEK, minimizing deformation [113]. Even under optimal conditions, scanbodies often fail to achieve perfect coaxiality with implants, leading to vertical and horizontal discrepancies in digital impressions [108].

#### 4.2.10. Implant Scan Body Bevel Location

The ISB is a precision-engineered device that is screwed onto a dental implant, and it serves as a connector between the implant and the 3D model. This device enables the scanner to accurately capture the 3D position of the implant and, thanks to its beveled design, also convey the orientation of the implant’s anti-rotational element. Research by Gómez-Polo et al. (2022) has demonstrated that a lingual orientation of this feature can significantly enhance both the trueness and precision of digital scans [3].

#### 4.2.11. Implant Scan Body Design

ISBs are recognized for their variability in design [115]. The geometry of ISBs influences the accuracy of digital impressions by features such as extensional structures and subtractive modifications can enhance accuracy, while sharp shapes and edgy surfaces may lead to inaccuracies [116,117,118]. Scan bodies with a flatter and simpler structure were linked to smaller deviations [55]. The optimal ISB height dimension appears to depend on the surrounding anatomical structures. When an implant is placed at the tissue level, allowing for complete visualization of the ISB, scanning accuracy tends to be higher. Conversely, subgingival implant placement can obscure a portion of the ISB, hindering accurate data capture. Some studies suggest that shorter ISBs may facilitate scanning in edentulous regions, while others propose that taller ISBs are preferable when adjacent to edentulous spaces [78,115,116,119]. The narrow-diameter ISBs, particularly those fabricated from PEEK, have been associated with increased accuracy [116,120]. If the occlusal surface of the ISB presents deficiencies, this can lead to loss of scanning accuracy [116]. Stimmelmayr et al. (2012) revealed that systematic error was reduced when scanning stone models than the polymer matted with scanning aid powder [107]. The material of ISBs influences both the scanning process and the accuracy of the resulting digital impression. Polyetheretherketone (PEEK) is a common choice due to its favorable scanning properties, but concerns remain regarding its potential for distortion and wear under clinical conditions [115,116,121]. Despite the use of ISBs appears to be promising, further research is necessary. Currently, limited scientific information is available regarding ISB and its influence on scan accuracy [115]. Further research is required to determine the optimal ISB design and its potential relationship with specific IOS technology for acquiring accurate intraoral digital implant scans [47].

#### 4.2.12. Implant Scan Body Retention System

There are various types of ISB retention systems, including screw-retained, Snap-On, and magnet-retained designs [47,78]. A notable gap exists in the literature regarding their influence on intraoral scanning accuracy [47]. Gómez-Polo et al. (2023) explicitly highlight the lack of studies investigating this relationship, emphasizing the need for further research [47]. It is mentioned that the magnetic retained system may be susceptible to movements induced by tongue pressure or contact with the IOS during scanning, potentially affecting the accuracy of digital data [3,78].

#### 4.2.13. Implant Scan Body Splinting Techniques

The splinting technique was developed to improve the accuracy of implant position transfer, enhancing prosthesis fit and reducing risks such as screw loosening, implant fracture, and bone resorption. It also minimizes discrepancies between the digital model and clinical situation, lowering the potential for human error and saving clinical time [122]. At least 17 splinting techniques have been reported, all employing a rigid connector between adjacent ISBs to enhance intraoral digitization and scanning accuracy. Mimicking the dental arch geometry, these devices facilitate full-arch digitalization by introducing curvature changes that improve scanner alignment [48,123]. Splinting solutions range from conventional materials like dental floss [124], light-cured resin, pattern resin, and PTFE tape to custom-made splints, assistive devices [122], and calibrated frameworks produced using combined additive and subtractive manufacturing techniques [125]. Splinting scan bodies or utilizing auxiliary apparatus generally enhances the trueness of digital implant impressions, particularly in complete-arch cases, by improving the stitching process and providing stable reference points, although effectiveness varies with clinical situation, scanning protocol, and IOS [122,126,127,128]. However, some studies reported no significant improvement or even negative effects of splinting on accuracy [122,129]. Overall, scientific evidence for complete-arch implant digital scans is still scarce [102,127,130,131]. Furthermore, in complete-arch cases with multiple implants and natural teeth, splinting scan bodies across a large span can amplify minor registration errors or discrepancies. In cases with severely undercut areas, deep subgingival margins, or other complex anatomical features, the rigidity of splinted scan bodies may hinder accurate capture [122]. Given the heterogeneity and limited number of existing studies, alongside the variability in IOS technologies and systems, identifying the most effective scan body splinting method for optimal digital implant scan accuracy remains challenging [48]. Additional research is required to develop standardized protocols and assess the long-term clinical performance of implant restorations fabricated from splinted scan body impressions [48,122].

Integrating the insights from the literature and the critical analysis of the presented findings, we can conclude that the factors influencing the accuracy of intraoral scanning of ISBs are largely analogous to those affecting intraoral scanning in general. This indicates that the accuracy of data acquisition principles in intraoral scanning is universally applicable across various contexts.

#### 4.2.14. Proximal Axial Wall Height (Height of the Prepared Tooth)

From practice, we know that for good retention, we need to provide an axial wall height of ≥4 mm [73]. Kim et al. 2024 found that the scanning accuracy decreases when the proximal axial wall height of the prepared tooth is ≥3.0 mm [73]. To overcome this aspect, one solution can be an IOS with a larger field of view [73].

#### 4.2.15. Inlay Preparation Design

The design of inlay preparations impacts the accuracy of intraoral scanning [28]. The depth of the occlusal cavity and the width of the gingival floor of the proximal box can influence both the trueness and precision of digital scans. Specifically, narrow and shallow preparations demonstrated the highest deviations, while wide and deep preparations yielded the most accurate results [28]. This may be attributed to improved scanner accessibility and better light reflection in deeper, wider preparations, which facilitate more complete data capture. Additionally, complex geometries with steep angles or sharp internal line angles may further challenge the scanner’s ability to accurately register surface details.

#### 4.2.16. Location of the Scanned Area

The distal surface of the prepared tooth showed less scanning accuracy, which suggests that interproximal regions, especially where the distance between adjacent teeth is small, pose a challenge for the IOS [32,84]. The mesial surface did not show similar levels of inaccuracy, this suggests that the distance between the marginal surface of the preparation and the adjacent tooth may be a factor in accuracy [84]. The buccal and lingual surfaces are more prone to consistent accuracy compared to the distal surface [84]. The molar region of the dental arch is particularly susceptible to greater inaccuracies [132]. The main causes of the molar region impressions are: the complex geometry of the dental arch, difficulty of access, saliva, crowding, and accumulation of merging errors [133]. In contrast, the anterior regions are generally easier to scan because of their accessibility and simpler geometry [31]. However, even in the anterior region, variations in tooth angulation and alignment can introduce errors if not properly addressed by the scanning technique [134].

#### 4.2.17. Arch Width

Arch width significantly influences the accuracy of intraoral scans, with wider arches presenting greater challenges for accurate data capture [36,94,135]. This is supported by Osnes et al. (2021), who demonstrated differences in arch width measurements between molars when using various IOSs [136]. Furthermore, the precision of digital impressions can be negatively impacted by arch width. This is attributed to the potential for merging errors during the creation of the 3D model from multiple scans [46,135]. The influence of arch width on accuracy appears to vary across different IOSs. Kaewbuasa and Ongthiemsak (2021) [135] observed distinct patterns of length deviation among scanners, with some consistently underestimating or overestimating arch width, while others exhibited variable performance depending on arch size. Similarly, angle deviations, indicative of image distortion, were also found to differ between scanners and arch widths [135]. These findings underscore the importance of considering arch width as a potential factor influencing the accuracy of intraoral scans. Clinicians should be aware of the potential limitations of different IOS technologies in capturing wider arches and exercise caution when interpreting scan data in these situations [32,36].

#### 4.2.18. Limited Mouth Opening

Within the limitations of in vitro conditions, mouth opening does not influence the accuracy of intraoral scanning [24]. It was revealed that there was no trueness and precision loss in the degree of mouth opening at 30, 37, and 40 mm [24]. This finding has implications, especially in pediatric dentistry, suggesting that IOSs can be reliably used in young patients. However, dentists should exercise caution when scanning patients with extremely limited mouth opening [24].

### 4.3. Intraoral Materials

#### 4.3.1. Material

The accuracy of scanning is influenced by the material itself and by the properties of the surface [64]. Dry, polished zirconia, with its similar reflectance characteristics to natural enamel, demonstrated optimal scanning performance [64]. However, the presence of moisture, especially at high levels, can compromise the accuracy of scans, particularly for materials like lithium disilicate [64]. Additionally, the color and translucency of materials, as well as the presence of existing dental restorations, can further impact the precision [64]. The materials can have different light reflectivity, which influences the scanning accuracy. For example, polycrystalline ceramic brackets, having less light reflectivity, showed higher precision than resin brackets [1,27]. Based on an evaluation of scanning trueness, composite, feldspathic ceramic, and zirconia demonstrated superior accuracy, with lithium disilicate glass–ceramic, enamel, and hybrid ceramic exhibiting intermediate levels [67]. Metal displayed the least accurate scanning results [67]. The polished surfaces result in increased light reflection and have a negative impact on scanning outcomes [64,67]. This underscores the critical role of surface type in scanning errors and suggests that scanner performance may vary with different dental or gingival surfaces and humidity retention on surfaces or other compounds [1]. The research on the effects of surface type and material on IOS accuracy is limited. Therefore, careful consideration of these factors is essential to optimize scanning accuracy [64].

#### 4.3.2. Material of the Bracket

In order to assess the progress of orthodontic treatment, it is a practice to conduct a scan of the dentition, including the brackets. Shin et al. (2021) [27] found that resin and resin-metal brackets, which exhibit reduced light reflection or absorption, result in higher precision, whereas greater errors occur when using other types of brackets, such as metal and ceramic. The translucency of the material may have contributed to this result [27].

#### 4.3.3. Translucency of the Materials

The outcomes from IOS are influenced by differences in restorative material and variations in tissue surface due to light scattering [137,138]. Scanning highly translucent materials results in a reduction in accuracy [12,66,137]. The restoration material type and IOS type significantly impacted scan data accuracy [139]. To mitigate these direct digital scanning issues, the application of powder prior to scanning has been suggested [140,141]. The accuracy of the scanning for enamel is inferior to that observed for dentin, and the presence of metallic surfaces is likely to exacerbate inaccuracies [12]. Translucency is a significant criterion in the selection of materials for dental restorations, as it contributes to the aesthetics and looks similar to natural dentition, and the accuracy could be improved by applying a scanning aid [66].

#### 4.3.4. Tooth Preparation Finishing Procedure

The findings of Revilla-León et al. (2023) [4] underscore the impact of tooth preparation finishing procedures on the accuracy of intraoral scanning. Their study revealed that air-particle abrasion techniques yielded the most precise and true results. This suggests that the choice of finishing procedure can directly impact the reliability of digital impressions, ultimately affecting the quality and success of restorative treatments [4].

#### 4.3.5. Tooth Color

The relationship between light and tooth structure affects color perception and the precision of intraoral scans. Tooth color can indicate the necessary lighting conditions for effective scanning. Darker teeth necessitate increased ambient lighting for improved visibility, particularly in the anterior mandibular area. In contrast, lighter teeth exhibit reduced sensitivity to lighting changes. These results underscore the necessity of considering both tooth color and lighting conditions to enhance the accuracy of intraoral scanning methodologies [72].

### 4.4. Ambient Conditions

#### 4.4.1. Lighting Conditions

Lighting conditions had a significant impact on both the trueness and precision of the digital scans [54]; varying the lighting condition could lead to a decrease in accuracy ranging from 7% to 43% [54]. The ambient light constitutes an essential factor for IOS, as it can introduce errors and delays in the scanning procedure [1,70]. Higher intensities of ambient light in the scanning room were associated with reduced scanning efficacy. This is likely due to the interference of external light sources with the scanner’s ability to accurately capture the surface details of the teeth and soft tissues [1], or sensor saturation due to excessively bright light and partial errors at points, and delays in data capture [70]. Different IOS systems may have varying sensitivities to light intensity. While some scanners perform optimally under brighter conditions, others might be more susceptible to overexposure, leading to inaccuracies in the scan data [2,54]. The color temperature or spectrum of the ambient light can also impact scan accuracy [70]. Certain wavelengths of light might interfere with the scanner’s ability to accurately capture the surface details of the teeth and soft tissues [2,54]. Different IOS technologies may exhibit different levels of susceptibility to ambient light variations. Some scanners might employ algorithms or filters to mitigate the effects of ambient light, while others might be more reliant on controlled lighting environments [1,2,33]. The specific scanning protocol used can influence the impact of ambient light. Factors such as scanning speed, scanning path, and the use of anti-reflective agents can all interact with ambient lighting conditions to affect the final scan accuracy [1,2,33]. In an investigation, Jivanescu et al. (2021) present a contradictory conclusion and assert that the environmental illumination possesses a restricted impact on the precision of intraoral scans, at a minimum for the IOS and conditions evaluated [57]. Clinically relevant light intensities, commonly found in dental offices, have minimal impact on IOS accuracy [57]. Overall, we have to be aware that a single ideal lighting condition for all IOSs does not exist. [2,33] and the clinicians should be aware of the optimal lighting recommendations for their specific IOS system and should consider using a light meter to ensure the optimal intensity and consistency across scans. Additionally, maintaining a clean scanning environment, using proper isolation techniques, and following the manufacturer’s recommended scanning protocols can further help mitigate the influence of ambient light and improve the accuracy of digital impressions [54].

#### 4.4.2. Humidity

Humidity negatively affects the accuracy of IOS. This effect is primarily attributed to the presence of saliva, a main source of moisture in the oral environment [33]. Saliva contamination leads to increased discrepancies in digital impressions compared to dry conditions [33], and the deviations measured in some saliva samples significantly exceeded the clinically acceptable threshold of 120 microns [33,64]. The presence of moisture (saliva) can significantly affect IOS accuracy due to the refraction of light in water during scanning, leading to increased deviation in angle measurements [27]. To minimize the negative impact of humidity on IOS accuracy, it is recommended to utilize tools like rubber dams, suction, and absorbent materials to effectively manage saliva and maintain a dry field throughout the scanning process [64] or dry tooth surfaces before scanning by employing compressed air or carefully wiping the surfaces with absorbent materials [64].

#### 4.4.3. Ambient Temperature

Clinicians should consider the potential impact of temperature fluctuations on IOS accuracy, particularly in environments with significant temperature variations [33]. Changes in ambient temperature can affect the accuracy of IOS [92]. Increasing the ambient temperature had a more significant positive effect on IOS accuracy than decreasing [92]; lowering the temperature did not negatively impact trueness, but it did affect precision [33]. Maintain a consistent and comfortable ambient temperature in the operatory phase [32], and allow the IOS to adjust to the room temperature before use. This can help prevent inaccuracies due to temperature differences between the device and the environment. Given the potential impact of ambient temperature variations on scanner accuracy, executing a calibration procedure when such fluctuations occur should be considered, as highlighted in the section on maintenance [72,92].

### 4.5. Scanning Strategy

#### 4.5.1. Scanning Strategy

According to Mai H.Y. and colleagues [63], the scanning strategy consists of all the movements of the scanner tip that are performed during the scanning procedure, the scan paths (straight or zigzag), the fact that the scanning area is continuous or segmented, and also the merging methods [63]. Gavounelis et al. (2022) [60] introduce several other factors in the definition of the scanning strategy, like starting point, rotation of the IOS, and speed of the movement. In the case of complete edentulous arches, the scanning strategy has an impact on accuracy, and this has to be chosen accordingly, and also with the maxillary or mandibular arch [71]. Each manufacturer of IOS devices recommends one or more specific scanning strategies. According to Gavounelis et al. (2022) [60], using alternative strategies not endorsed by the manufacturer may increase the risk of reduced accuracy. The scanning strategy affects the accuracy differently depending on the type of the IOS, and to achieve optimal results, the scanning strategy should be adjusted to match the characteristics of each device [60]. The scanning strategy is as important as the scanned area is more extensive, because the chances of errors are proportionally increased, and the segmental scan seems to be more accurate than a full arch scan [63]. The strategy, starting with teeth and moving to edentulous ridges, might be most effective in some situations [65]. In the case of segmentation, the strategy has to be conceived in accordance with the stitching software recommendations, and the two segmented scans are more accurate than a full arch and also than a three segmented scan [63]. Regarding implant scanning, a two-step scanning approach—initially capturing the crest without scan bodies, followed by a subsequent scan with scan bodies in place and then superimposing the images—yields significantly lower precision compared to a direct scanning strategy with scan bodies already affixed [55]. Manufacturers of IOSs often provide recommendations for scanning strategies, particularly for single crowns, quadrants, and complete arch impressions [32,37]. A comprehensive understanding of each component that is detailed below will facilitate the formulation of a scanning strategy optimized for the unique constraints encountered in each clinical situation.

#### 4.5.2. Scanning Origin

Establishing the scanning origin involves considering a combination of factors related to the morphology, intraoral materials, and conditions. Oh et al. (2020) [142] noted that the starting position of the scan does not affect the scanning accuracy. Regarding this statement, we have to keep in mind that the study was made on a complete edentate arch, and this is almost a singular opinion in the area [142]. On the other hand, there is a consensus that starting on the occlusal surface of a molar is recommended [36,37,60,64,86,143,144,145]. The occlusal-first scanning pattern showed higher precision than the S-shaped scanning pattern [86]. From this point forward, the starting point depends on the mouth configuration. For better accuracy, it is recommended to start the scan from the end of the remaining dental arch, but not from an isolated tooth towards a mucosal area [61]. Increased distance from the scanning origin can diminish accuracy, and adhering to some manufacturer recommendations to position the scanning origin at the most posterior region of the dental arch may not consistently yield optimal results [146]. The complexity of the scanned surface impacts the stitching process. Starting a scan in an area with complex morphology, like the occlusal surfaces of posterior teeth, can improve the accuracy of image stitching compared to starting on simpler surfaces, such as the buccal surfaces [60].

In the case of a completely edentulous maxilla, the buccopalatal (BP) technique proved to be most accurate [100]. In this technique, the scan begins on the ridge top side of the edentulous arch, starting from the left maxillary tuberosity, proceeding longitudinally along the ridge, and ending at the right tuberosity. It then continues on the buccal side and finally on the palatal vault. The palatal vault is initially scanned with a counterclockwise movement and then with a longitudinal movement to close the gap along the midline of the palate [95]. A better but not statistically significant improvement in accuracy is reported when the palate was scanned versus when not scanned [147]. The beginning of scanning on the palatal surfaces leads to increased accuracy due to the presence of more distinct anatomical features and rigid overlapping [145].

#### 4.5.3. Scanning Distance

Scanning distance is the length of a continuous scan or the linear distance covered in a single, uninterrupted sweep of the scanner, such as in a full-arch scan where the scanner moves from one end of the arch to the other. Longer scanning distances might require more image stitching and potentially introduce greater cumulative errors [33,36,53]. There is a difference in accuracy between short-arch scans and complete-arch scans. For example, the complete-arch scans, covering a larger distance, pose greater challenges for IOS devices in terms of accuracy, especially for edentulous areas [9,53,56]. Furthermore, maxillary scans displayed higher error rates than mandibular scans. In the full arch scans, the posterior teeth have greater errors than the anterior teeth, and the right posterior teeth within mandibular scans manifested a more pronounced variability than their left counterparts [56].

#### 4.5.4. Scanned Arch

The anatomical characteristics of the scanned arch significantly influence the precision of intraoral scans, while having a negligible effect on their trueness. The larger surface area of the maxillary arch compared to the mandibular arch might suggest a potential advantage in terms of accuracy, and at the same time, no difference in trueness [71]. However, the mandibular arch showed superior precision, likely due to the increased amount of data points captured during the scanning process. These findings underscore the importance of considering the specific anatomical features of the scanned arch when interpreting the accuracy of digital impressions, particularly in the context of completely edentulous patients [71].

#### 4.5.5. Landmarks

IOSs may be less accurate when scanning wider areas and smooth surfaces like an edentulous jaw [15,33] or in the posterior region [15]. Using artificial landmarks enhances both trueness and precision [33]. These landmarks are physical markers placed on teeth or tissues [33] or healing abutments [9], providing extra reference points for the scanning software [9,15,33]. Artificial landmarks can also address issues with stitching errors that arise when there are large gaps between scanned segments [15]. Strategically placed landmarks can enhance the alignment of scan data, leading to a more accurate digital model [33]. Placing resin markers on the palate of edentulous patients is recommended for full-arch scans. This helps overcome the challenges posed by the lack of teeth and the presence of moving tissues like the tongue [33]. Landmarks are crucial for precise alignment during the bite registration process in oral scanning [9] and help to reduce stitching errors that are more likely to occur on flat surfaces with fewer irregularities [15]. Healing abutments can be used as a landmark structure. Jin et al. (2021) validated in their study that healing abutments facilitate the accuracy of scanning in edentulous areas [9]. Healing abutments introduce a geometric complexity which facilitates the IOS detection and the digital image to be stitched smoothly [9].

#### 4.5.6. Scanning Sequence

The scanning sequence after Diker and Tak (2020) [37] is considered the chronology of the scanning movements. The scanning sequence is a subfactor of the scanning strategy, focusing on the precise order of scanning different oral segments [37,65]. Chang et al. (2023) introduce the term sequential range, which represents a subdivision of the scanning path that is performed in one time [65]. The scanning sequence significantly impacts the quality of the digital impression, as an unoptimized sequence can lead to stitching errors, data distortion, and increased image noise. Following a consistent and validated scanning sequence helps improve data alignment and continuity, particularly in full-arch scans. Additionally, optimized sequencing minimizes the likelihood of missed areas and rescans, ultimately enhancing both efficiency and scan accuracy.

#### 4.5.7. Scanning Pattern

The scanning pattern or scanning path, the route employed with an IOS, is a factor that can affect the accuracy of digital impressions [148] and play an important role in obtaining accurate results with IOSs. While manufacturers recommend specific scan patterns, evidence supporting the superiority of these patterns is limited [148]. This relationship varies between different IOS models and their image merging software [148]. While some scanners are more affected, other systems showed no significant differences in trueness among the different scan patterns [148]. Erratic movements of the IOS head can disrupt the stitching process, reducing accuracy [60]. Maintaining a stable rotational and vertical position of the IOS head is crucial for accurate results [142,149]. A scan path with the shortest distance between the start and end points yields the highest accuracy [75]. Following the manufacturer’s guidelines for the specific IOS is recommended, as these guidelines are often based on comprehensive testing and optimization [33,60].

#### 4.5.8. Scanning Height

According to Rotar et al. (2022) [62], the optimal accuracy in intraoral scanning is achieved at a distance of 10 mm between the scanning tip and the prepared area. Accuracy decreases with deviations from this optimal distance, as both trueness and precision values are compromised by increasing the scanning distance beyond 10 mm, with the poorest accuracy observed at 23 mm [62]. Furthermore, accuracy is also diminished at very close scanning distances of 5 mm [62]. In a study on three IOS models, Kim et al. (2019) concluded that the best accuracy is between 2.5 and 5 mm height [134]. Button et al. (2024) tested four IOS brands and found that the maximum accuracy is between 0 and 4 mm in distance [149]. Furthermore, the study by Amornvit et al. (2021) supports the notion that greater scanning distances generally lead to decreased accuracy across various IOSs, emphasizing the importance of maintaining an optimal distance during scanning [150]. According to Button et al. (2024), we can conclude that the optimal scanning distance (height) for accuracy is not a universal value; it is specific to the IOS being used, and clinicians should consider these device-specific findings when using or acquiring intraoral scans [149]. In essence, regardless of the specific IOS, achieving accurate digital impressions hinges on an adequate depth of field (DOF). Consequently, a sufficient DOF is imperative for precise reproduction of edge sharpness, though exceeding it can compromise image accuracy [28].

#### 4.5.9. Scanning Speed

It is accepted that elevated scanning speed can compromise the accuracy of the scan because the IOS may not capture enough data points for an accurate 3D model reconstruction. On the other hand, scanning at an excessively reduced pace could increase the risk of patient movement or fatigue, which can also negatively affect the accuracy of the scan. It is recommended to follow the manufacturer’s guidelines for scanning speed to ensure optimal results [33].

#### 4.5.10. Scanning-Aid

Scanning-aid materials are addressing the challenges associated with scanning: deep vertical preparations and metallic restorations can pose challenges for IOS due to light reflection [22,33], translucent ceramic materials, such as zirconia, lithium disilicate glass–ceramic, and leucite-reinforced glass–ceramic, exhibit a phenomenon called subsurface scattering, where light penetrates the surface and scatters within the material before exiting [66] and the IOS’s limited ability to capture accurate data in difficult-to-scan areas [33]. Powder coatings were initially popular but have limitations in achieving uniform application, especially in the presence of saliva or limited inter-arch space. Factors like operator skill and tongue movement can affect powder application consistency [33,59]. Different application distances and times can result in variations in the coating thickness, leading to scan errors [59]. Liquid-type agents offer a more controlled and uniform application compared to powder sprays. Liquid agents are particularly beneficial in creating thinner layers, which can contribute to increased accuracy [33]. Liquid-type scanning-aid agents can be seen as a preferable alternative to powder-based materials [33]. These agents, applied using a brush technique, offer several advantages, such as more controlled and uniform application. This leads to a more consistent coating thickness, minimizing potential scan errors and eliminating particle scattering. This addresses the respiratory concerns associated with powder sprays [59]. Using scanning-aid materials improves accuracy by enhancing surface opacity and reducing reflections. Scanning-aid materials contribute to more precise and reliable digital impressions [33,59,66]. Liquid-type agents have been shown to be effective in facilitating the scanning of narrow and deep areas, as well as metallic restorations [33]. With scanning aid, the scanning time is reduced [59,66], and full-arch scanned images can be obtained more efficiently [59].

#### 4.5.11. Scanning Protocol

The scanning protocol is the set of specific guidelines for scanning strategy, setup, conditions, and scanner calibration, often outlined by the manufacturer, to ensure accuracy and minimize errors in digital impressions [65,151]. It is important to consider the scanning protocol prescribed by the manufacturer to achieve optimal accuracy [152].

#### 4.5.12. Experience of the Operator (Training and Age)

While experienced dental professionals can complete intraoral scans more quickly, it seems that their expertise does not necessarily lead to more accurate results [10,17]. Studies have shown that even less experienced operators can produce scans of very high accuracy [10,17]. Remarkably, dedicating additional time to the scanning process, particularly for an inexperienced operator, can yield more accurate outcomes, especially in edentulous regions [17]. Training significantly improves scanning accuracy across operators, regardless of age or prior experience [68]. The improvement is more pronounced among inexperienced users [68]. Despite overall training gains, older inexperienced operators did not reach the same level of accuracy as their younger or experienced counterparts, suggesting that age may influence the effectiveness of training for certain IOS technologies [68].

#### 4.5.13. Patient Preparation

While patient preparation for intraoral scanning has not been identified as an independent factor directly influencing precision, the procedural steps necessary for effective patient preparation management can be delineated based on the factors discussed herein. It is, therefore, imperative that patient preparation constitute the initial phase in the implementation of any scanning strategy and involves several key steps to ensure accurate digital impressions [32]. Maintaining a clean oral environment involves ensuring the teeth are free of debris and plaque [22]. The scanning target must be visually confirmed by the scanner; therefore, controlling saliva and other oral fluids has to be completed, as they can interfere with the optical scanning process [14,36]. Adequate soft tissue management, including gingival displacement, is necessary to clearly expose the preparation finish line, especially for subgingival preparations [22,153]. Gingival retraction cords or materials may be used to achieve this [22]. Proper soft tissue control and retraction are critical for an accurate optical scan [153]. Clinicians should aim for smooth and regular preparation surfaces with rounded internal line angles, avoiding sharp edges, grooves, and boxes, as these are easier for light scanners to capture [36]. Sudden changes in curvature can lead to greater deviations [36]. Ensuring patient comfort is important to minimize movement during the scanning process [16,154].

#### 4.5.14. Limitations

The limitations of in vitro studies in this research stem from the fact that they do not fully replicate real-world clinical conditions, which can impact the generalizability of the findings. Unlike in vivo studies, in vitro experiments lack dynamic oral factors, such as saliva, blood, soft tissue movement, and patient-related variables like involuntary motion or limited mouth opening, which can all affect intraoral IOS accuracy [155,156]. Additionally, lighting conditions and humidity levels in a controlled laboratory setting differ significantly from those encountered in a clinical environment, potentially leading to overestimated accuracy results [157]. Another limitation is that artificial models and materials used in vitro may not perfectly mimic the optical and structural properties of natural dentition and soft tissues, leading to differences in scanning precision [158]. Furthermore, the absence of patient-related anatomical constraints means that scanner maneuverability and operator technique may not be tested under real clinical challenges [159]. Despite these limitations, in vitro studies remain valuable for controlled comparisons of different scanning strategies, IOS models, and influencing factors, but their findings must be cautiously interpreted when applied to clinical practice.

Another limitation of this research is the wide variety of IOS brands and scanning technologies available on the market, which introduces challenges in standardization and comparability. Different IOS systems utilize various scanning technologies, such as confocal imaging, active triangulation, optical coherence tomography, and active wavefront sampling, each with unique strengths and limitations that can influence accuracy [160]. Additionally, manufacturers frequently update their hardware and software, leading to variations in performance across different versions of the same IOS model [161]. The lack of a universal scanning protocol further complicates direct comparisons, as different scanners may require specific scanning paths, speeds, or calibration procedures to achieve optimal results. These technological disparities mean that results obtained with one IOS system may not be fully applicable to another, limiting the generalizability of findings [149]. Although this study aimed to identify common influencing factors rather than evaluate specific brands, the variability in scanning technology remains a challenge, and further research is needed to establish standardized protocols that ensure consistent accuracy across different IOS models.

Another significant limitation in intraoral scanning is the restriction of the scanner tip within the oral cavity, which can impact the accuracy and completeness of digital impressions [162]. Unlike in vitro conditions, where scanners have unrestricted access to models, in clinical settings, the size and shape of the scanner tip can limit maneuverability, particularly in posterior regions, patients with small oral openings, or cases with anatomical obstructions. These restrictions can lead to difficulty in capturing deep marginal lines, areas with high tissue mobility, or edentulous ridges, where scanning precision is crucial [155]. Additionally, certain scanner tips may require specific angulations to properly capture the scanned area, increasing the risk of errors if the device cannot be positioned correctly [163,164]. The scanner’s field of view also plays a role, as larger scanner heads can improve precision but may struggle to access tight interproximal spaces, while smaller tips enhance accessibility but may compromise trueness due to a narrower imaging range and increased stitching errors. These clinical challenges underscore the need for practitioners to select IOS systems with ergonomic designs that balance accessibility and accuracy, while also refining scanning techniques to mitigate the impact of these physical restrictions [165].

Another important limitation in intraoral scanning is the influence of anatomical structures, musculature, and the surface characteristics of teeth and restorations on scanning accuracy [166]. Unlike in vitro conditions, where motionless models are used, in a clinical setting, soft tissues such as the tongue, lips, and cheeks can interfere with scanning, causing involuntary movement and distortions in the digital impression. The buccal mucosa and floor of the mouth, in particular, are highly mobile, making it difficult to achieve consistent, distortion-free scans, especially in edentulous patients [165]. Additionally, tooth surface roughness and the type of restorative material can significantly impact scanning precision. Highly polished surfaces, such as glazed ceramics or metal restorations, reflect light excessively, leading to scanning errors, while porous or rough surfaces may introduce inaccuracies due to irregular reflections and scattering of the scanner’s optical signals [167]. Translucent materials, such as lithium disilicate or zirconia restorations, further complicate the scanning process due to subsurface light scattering, which can reduce trueness [168]. These factors highlight the clinical challenges of achieving accurate intraoral scans, emphasizing the need for proper isolation techniques, optimized scanning strategies, and advancements in scanner technology to improve accuracy in complex intraoral environments.

#### 4.5.15. Procedural Guideline Proposal

**Figure 3:**
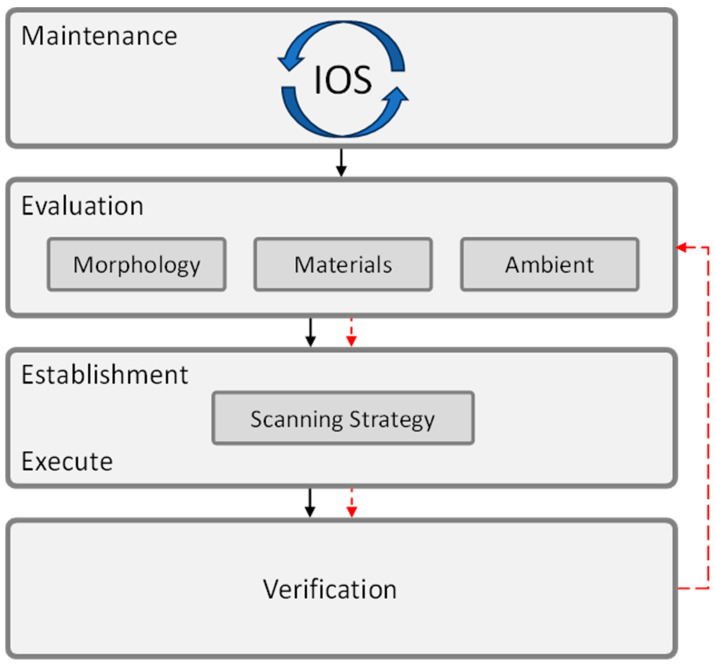
The four-step procedural guideline for enhanced intraoral scanning accuracy.

To ensure high-quality intraoral digital impressions and to achieve the highest possible accuracy with the intraoral scanner available in the facility, we have considered the 43 influencing factors identified and systematically categorized in the five distinct groups. With the aim of providing the practitioner with a straightforward and intuitive guideline, we propose a four-step protocol (Figure 3) to be followed throughout the intraoral scanning process. This structured approach is intended to optimize scanning outcomes by integrating evidence-based recommendations derived from the comprehensive analysis of the identified variables. The proposed guideline is designed to address the most common challenges encountered in clinical settings and is applicable across all intraoral scanner systems, irrespective of brand or model.

Maintenance consists of regularly checking for software updates for both the intraoral scanner and the associated CAD/CAM system. Updates can enhance scanning accuracy and system stability. Evaluate whether updates are necessary and integrate the update process into the regular maintenance routine to ensure optimal performance. Calibrate the IOS according to the manufacturer’s recommendations, ideally before each scanning session, especially if ambient temperature changes. Follow the specific calibration protocol for your scanner model and store calibration tools in a stable environment.

Evaluation of morphology, materials, and ambient—before initiating intraoral scanning, a comprehensive evaluation of patient-specific anatomical and clinical conditions is essential to anticipate potential scanning challenges. This includes assessing dental crowding, tooth inclination, edentulous areas, palatal morphology, inter-tooth and inter-implant distances, as well as implant-related variables such as position, depth, angulation, and scan body characteristics (fit, design, bevel location, retention system, and splinting technique). Additionally, evaluate preparation parameters like proximal axial wall height, inlay design, and the location of the scanned area. Consider arch width and limitations in mouth opening that may restrict scanner access. Material-related factors, including type, translucency, and color, should also be reviewed, alongside the finishing of tooth preparations. Environmental conditions such as lighting, humidity, surface wetness, and ambient temperature must be controlled to ensure optimal scan accuracy.

Establish and execute the scanning strategy—based on the outcomes of the evaluation step, the practitioner determines and implements a scanning strategy tailored to the specific clinical scenario. This strategy encompasses multiple parameters, including the preparation of the scanned area, the selection of the scanning origin, optimal scanning distance, the specific arch to be scanned, identification of anatomical landmarks, and the definition of scanning sequence and pattern. Additionally, the practitioner must establish the appropriate scanning height and speed, consider the use of scanning aids if necessary, and adhere to a standardized scanning protocol to ensure accuracy and consistency in digital impression acquisition.

Verification—post-scan verification is essential to ensure accuracy and completeness. Operators should review the digital model in real-time, identifying gaps, distortions, or missing data, and perform selective rescanning of problematic areas rather than restarting the scan entirely.

By adhering to this procedural guideline, dental practitioners can achieve consistent, high-accuracy intraoral scans regardless of the IOS brand or technology used, ensuring reliable digital impressions for prosthetics, orthodontics, and implantology.

## 5. Conclusions

This study highlights the critical factors influencing the accuracy of IOS and establishes a procedural guideline to optimize scanning outcomes, regardless of the specific IOS brand or technology used. By systematically analyzing the factors that impact the scanning accuracy, we have identified a procedural guideline that ensures the conditions for high trueness and precision in intraoral digital impressions. The results emphasize that accuracy is not solely dependent on the IOS device itself but is significantly influenced by how the scanning process is executed.

Future research should focus on developing standardized evaluation metrics for IOS performance across different clinical scenarios, as well as advancing software algorithms and hardware improvements to further enhance scanning accuracy. While this study provides a comprehensive framework for optimizing intraoral scanning, ongoing innovation and refinement in scanning techniques will continue to shape the future of digital dentistry, ensuring greater precision, efficiency, and reliability in patient care.

## Figures and Tables

**Table 1:** Risk of Bias Assessment of Observational Studies According to NOS Criteria.

Author	Criterion 1	Criterion 2	Criterion 3	Criterion 4	Criterion 5	Criterion 6	Criterion 7	Criterion 8	Criterion 9	Total Score
Braian M. et al. [53]	1	1	1	1	1	1	1	1	1	9
Revilla-León M. et al. [54]	1	1	1	1	1	1	1	1	1	9
Revilla-León M. et al. [2]	1	1	1	1	1	1	1	1	1	9
Martínez-Rodríguez C. et al. [1]	1	1	1	1	1	1	1	1	1	9
Diker B. and Tak Ö. [37]	1	1	1	1	1	1	1	1	1	9
Motel C. et al. [55]	1	1	1	0	1	1	1	1	1	8
Moon Y.-G. and Lee K.-M. [56]	1	x	1	x	1	1	1	1	1	7
Schimmel M. et al. [17]	1	1	1	1	1	1	1	1	1	9
Jivanescu A. et al. [57]	1	x	1	1	1	1	1	1	1	8
Jin G. et al. [9]	1	1	1	1	1	1	1	1	1	9
Lee Y.-C. et al. [58]	1	x	1	1	1	1	1	x	1	7
Shin S.-H. et al. [27]	1	1	1	1	1	1	1	1	1	9
Lim J.-H. et al. [12]	1	x	1	1	1	1	1	x	1	7
Oh H.-S. et al. [59]	1	x	1	1	1	1	1	1	1	8
Gómez-Polo M. et al. [3]	1	1	1	1	x	1	1	1	1	8
Gavounelis N.A. et al. [60]	1	1	1	1	1	1	1	1	1	9
Kim E.-Y. et al. [61]	1	x	1	1	1	1	1	1	1	8
Rotar R.N. et al. [62]	1	1	1	1	1	x	1	1	1	8
Mai H.Y. et al. [63]	1	1	1	1	1	1	1	1	1	9
Revilla-León M. et al. [48]	1	1	1	1	1	1	1	1	1	9
Agustín-Panadero R. et al. [64]	1	1	1	1	1	1	1	1	1	9
Chang I.-C. et al. [65]	1	x	1	x	1	1	1	1	1	7
Cho J.-H. et al. [66]	1	1	1	1	1	1	1	1	1	9
Park Y. et al. [28]	1	x	1	1	x	1	1	1	1	7
Shimizu T. et al. [15]	1	x	1	1	1	1	1	1	1	8
Thomas A.A. and Jain R.K. [10]	1	1	1	x	1	1	1	1	1	8
Elter B. and Tak Ö. [67]	1	x	1	1	1	1	1	1	1	8
Zarauz C. et al. [68]	1	x	1	1	1	1	1	1	1	8
Sorrentino R. et al. [69]	1	x	1	1	1	1	1	1	1	8
Karakuzu M. et al. [70]	1	1	1	1	1	1	1	1	1	9
Jamjoom, F.Z. et al. [71]	1	x	1	1	1	1	1	1	1	8
Zhou Y. et al. [72]	1	x	1	1	1	1	1	1	1	8
Ye J.R. and Jain R.K. [24]	1	1	1	1	1	1	1	x	1	8
Jamjoom F.Z. et al. [71]	1	1	1	1	1	1	1	1	1	9
Kim S.-Y. et al. [73]	1	1	1	1	1	1	1	1	1	9
Liu C.-T. et al. [74]	1	x	1	x	1	1	1	1	1	7
Choi E.-J. et al. [75]	1	x	1	1	1	1	1	1	1	8

C1—Representativeness of the exposed cohort; C2—Selection of the non-exposed cohort; C3—Ascertainment of exposure; C4—Demonstration that outcome was not present at start; C5—Comparability of cohorts on the basis of design or analysis; C6—Control for additional factors; C7—Assessment of outcome; C8—Was follow-up long enough for outcomes to occur?; C9—Adequacy of follow-up.

**Table 2:** Risk of Bias Assessment of Systematic Reviews Based on AMSTAR 2 Criteria.

Author (Citation)	C1	C 2	C3	C 4	C 5	C 6	C 7	C 8	C9	C 10	C 11	C 12	C 13	C 14	C 15	C 16	Total Score
Hardan L. et al. [33]	1	x	1	1	1	1	1	1	1	x	1	1	1	1	1	1	14
Revilla-León M. et al. [4]	1	x	1	1	1	1	1	1	1	1	x	1	1	1	x	1	13
Gehrke P. et al. [76]	1	1	1	1	1	1	1	1	1	x	x	1	1	1	x	1	13

C1—Clear research question and inclusion criteria; C2—Protocol registered before review commencement; C3—Explanation of study selection; C4—Comprehensive literature search; C5—Study selection in duplicate; C6—Data extraction in duplicate; C7—List of excluded studies with justification; C8—Description of included studies; C9—Risk of bias assessment of individual studies; C10—Funding sources of included studies reported; C11—Appropriate methods for statistical combination of results; C12—Impact of risk of bias on the results; C13—Consideration of risk of bias when discussing findings; C14—Explanation of heterogeneity; C15—Investigation of publication bias; C16—Declaration of conflict of interest.

**Table 3:** Identified factors that influence the accuracy of IOS by article.

No.	Authors	Year	Vivo/ Vitro	Factors
1.	Braian M. et al. [53]	2019	Vitro	Scanning distance
2.	Revilla-León M. et al. [54]	2020	Vivo	Lighting conditions
3.	Diker B. and Tak Ö. [37]	2020	Vitro	Scanning sequence
4.	Revilla-León M. et al. [2]	2020	Vitro	Lighting conditions
5.	Martínez-Rodríguez C. et al. [1]	2020	Vitro	Lighting conditions
				Surface material
				Crowding of teeth
				Inclination of teeth
6.	Motel C. et al. [55]	2020	Vitro	Scanning strategy
				Implant scan body (design)
7.	Moon Y.-G. and Lee K.-M. [56]	2020	Vivo	Scanning distance
8.	Lim J.-H. et al. [12]	2021	Vitro	Translucency of the materials
9.	Jin G. et al. [9]	2021	Vitro	Healing abutments as landmarks
				Number of missing teeth (edentulous condition)
10.	Schimmel M. et al. [17]	2021	Vitro	The experience of the operator
11.	Jivanescu A. et al. [57]	2021	Vitro	Lighting conditions
12.	Lee Y.-C. et al. [58]	2021	Vitro	Edentulous condition
13.	Oh H.-S. et al. [59]	2021	Vitro	Scanning-aid materials
14.	Shin S.-H. et al. [27]	2021	Vitro	Material of the bracket
15.	Kim E.-Y. et al. [61]	2022	Vitro	Scanning origin
16.	Mai H.Y. et al. [63]	2022	Vitro	Scanning strategy
17.	Gómez-Polo M. et al. [3]	2022	Vitro	Implant scan body bevel location
				Implant angulation
				Implant position
18.	Rotar R.N. et al. [62]	2022	Vitro	Scanning distance (height)
19.	Gavounelis N.A. et al. [60]	2022	Vitro	Scanning strategy
20.	Shimizu T. et al. [15]	2023	Vitro	Landmarks
21.	Elter B. and Tak Ö. [67]	2023	Vitro	Adjacent substrate (material)
22.	Revilla-León M. et al. [48]	2023	-	Lighting conditions
				Scanning pattern
				Implant scan body design
				Implant scan body splinting techniques
				Arch location (scanned arch)
				Implant position
				Inter-implant distance
				Implant depth
				Implant angulation
				Interdental space (inter-tooth distance)
23.	Zarauz C. et al. [68]	2023	Vivo	Age of operator
				Training of operator (experience)
				Software version
24.	Chang I.-C. et al. [65]	2023	Vitro	Scanning protocol
25.	Hardan L. et al. [33]	2023	-	Scanning speed
				Scanning pattern
				Landmarks
				Humidity
				Scanning-aid (material and agents)
				Lighting conditions
				Scanning distance
				Ambient temperature
				Software version
				Scan resolution
				Size of the scanner head
				Custom abutment library data
26.	Cho J.-H. et al. [66]	2023	Vitro	Translucency of ceramic restorative materials
				Scanning aid
27.	Thomas A.A. and Jain R.K. [10]	2023	Vivo	The experience of the operator
28.	Agustín-Panadero R. et al. [64]	2023	Vitro	Wetness of the surface
				Materials
29.	Park Y. et al. [28]	2023	Vitro	Inlay preparation design
30.	Revilla-León M. et al. [4]	2023	-	Tooth preparation finishing procedure
31.	Kim S.-Y. et al. [73]	2024	Vitro	Inter-tooth distance
				Proximal axial wall height
32.	Choi E.-J. et al. [75]	2024	Vitro	Scan path (scanning pattern)
33.	Gehrke P. et al. [76]	2024	-	Implant scan body design, type of ISB
				Implant scan body material (materials)
				Implant scan body fit
				Implant position
				Implant angulation
				Operator skill (the experience of the operator)
				Scanning strategy
				Scanning aids
34.	Zhou Y. et al. [72]	2024	Vitro	Tooth color
				Lighting conditions
35.	Karakuzu M. et al. [70]	2024	Vitro	Lighting conditions
36.	Liu C.-T. et al. [74]	2024	Vitro	Scanning strategy
37.	Sorrentino R. et al. [69]	2024	Vitro	Palatal morphologies
38.	Jamjoom F.Z. et al. [71]	2024	Vitro	Scanning strategy
				Scanned arch
39.	Jamjoom F.Z. et al. [77]	2024	Vitro	Scanning aid (liquid-type)
40.	Ye J.R. and Jain R.K. [24]	2024	Vitro	Limited mouth opening

**Table 4:** Additional factors identified in reviews not included in initial selection of articles.

No.	Authors	Year	Vivo/ Vitro	Factors
1.	Alkadi L. [32]	2023	-	Location of the scanned area
				Arch width
				Scan resolution
				Software versions and updates
				Scanner head size
2.	Gómez-Polo M. et al. [47]	2023	-	Implant scan body retention system
3.	Revilla-León M. et al. [78]	2021	-	Implant scan body design

**Table 5:** The factors structured into the five groups.

**IOS—characteristics and maintenance**	1.	Software versions and updates [32,33,68]
	2.	Scan resolution [32,33]
	3.	Size of the scanner head [32,33]
	4.	Custom abutment library data [33]
**Intraoral morphology**	5.	Crowding of teeth [1]
	6.	Inclination of teeth [1]
	7.	Edentulous condition [9,58]
	8.	Palatal morphologies [69]
	9.	Inter-tooth distance [73]
	10.	Inter-implant distance [48]
	11.	Implant position [3,48,76]
	12.	Implant depth [48]
	13.	Implant angulation [3,48,76]
	14.	Implant scan body fit [76]
	15.	Implant scan body bevel location [3]
	16.	Implant scan body design [48,55,76,78]
	17.	Implant scan body retention system [47]
	18.	Implant scan body splinting techniques [48]
	19.	Proximal axial wall height [73]
	20.	Inlay preparation design [28]
	21.	Location of the scanned area [32]
	22.	Arch width [32]
	23.	Limited mouth opening [24]
**Intraoral materials**	24.	Materials [1,27,64,67,76]
	25.	Translucency of the materials [12,66]
	26.	Tooth preparation finishing procedure [4]
	27.	Tooth color [72]
**Ambient conditions**	28.	Lighting conditions [1,2,33,48,54,57,70,72]
	29.	Humidity, wetness of the surface [33,64]
	30.	Ambient temperature [33]
**Scanning strategy**	31.	Scanning strategy [55,60,63,71,74,76]
	32.	Scanning origin [61]
	33.	Scanning distance [33,53]
	34.	Scanned arch [48,71]
	35.	Landmarks [9,15,33]
	36.	Scanning sequence [37]
	37.	Scanning pattern [33,48,75]
	38.	Scanning height [62]
	39.	Scanning speed [33]
	41.	Scanning-aid [33,59,66,76,77]
	42.	Scanning protocol [65]
	43.	Experience of the operator (training and age) [10,17,68,76]

## Data Availability

The research was registered at Open Science Framework (OSF) and can be found under registration DOI https://doi.org/10.17605/OSF.IO/N8PWD. All the metadata were uploaded and are available under the registration code osf.io/n8pwd.
